# Supplementary material for: Comparable Vδ2 Cell Functional Characteristics in Virally Suppressed People Living with HIV and Uninfected Individuals
Source: Cells. 2020 Dec 1;9(12):2568. doi: 10.3390/cells9122568 (PMC7760715; doi:10.3390/cells9122568)
Supplement: Supplementary file 1 [file cells-09-02568-s001.pdf]

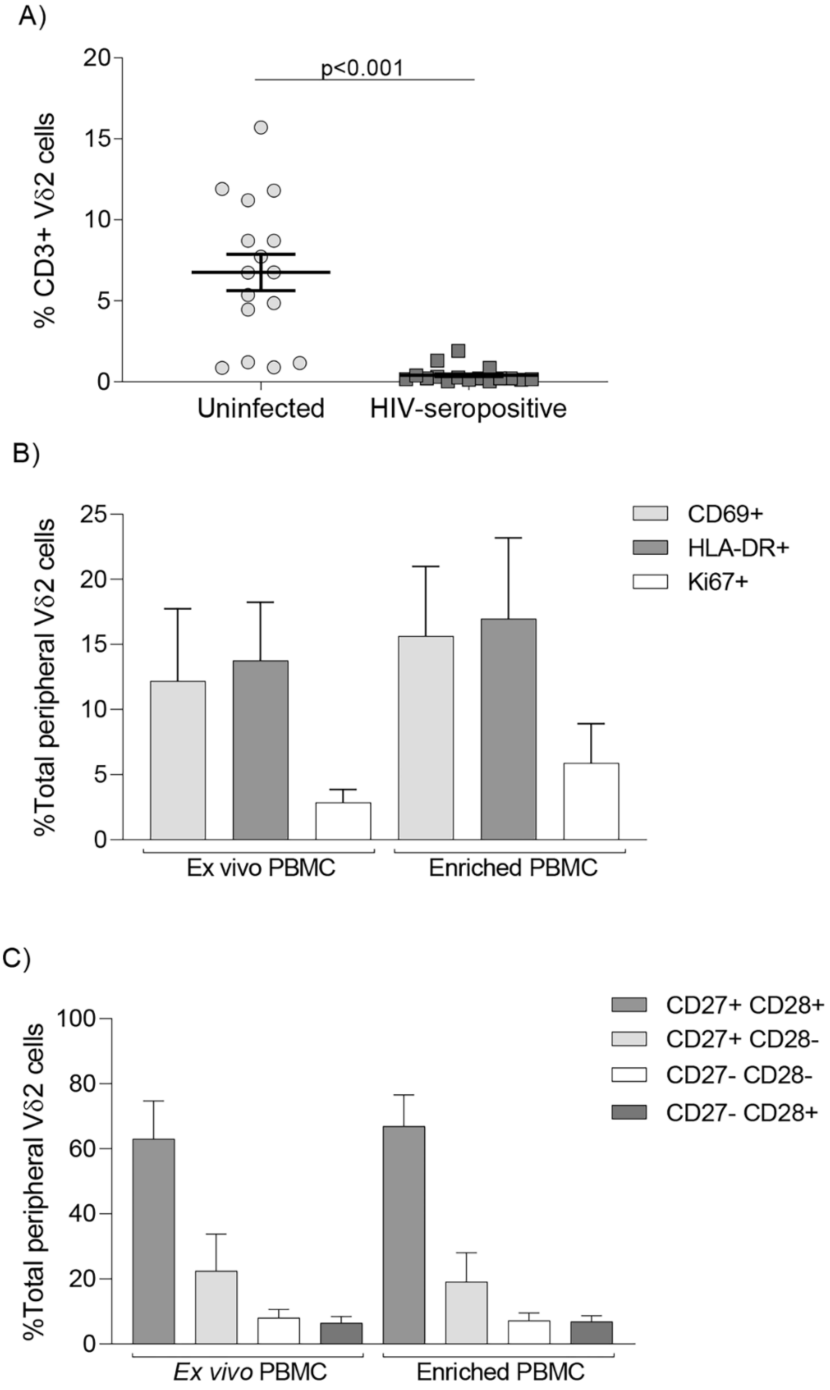

Figure S1: Ex vivo frequency of Vδ2 cells and comparison of ex vivo and magnetically enriched subpopulations.

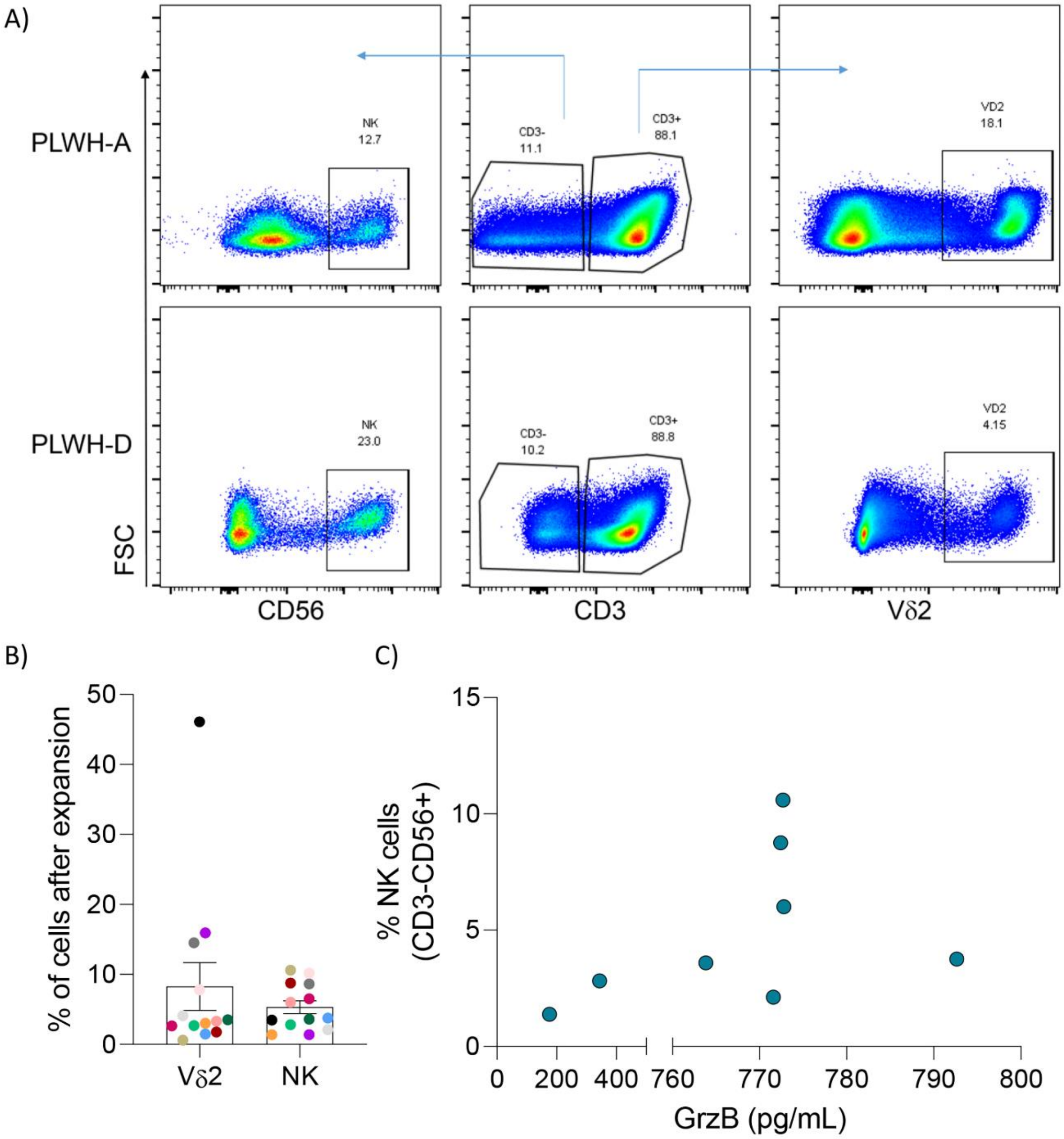

Figure S2: Analysis of Vδ2 and NK cell frequency in PBMC cultures from PLWH exposed to PAM and IL-2 for seven days. (A) Representative pseudocolor plots showing the frequency of Vδ2 cells (right) and NK cells (left). Example of one individual with high (up panels) or low (down panels) Vδ2 expansion. (B) Frequency of Vδ2 cells and NK cells. (C) Lack of correlation between the frequency of NK cells and GrzB production.

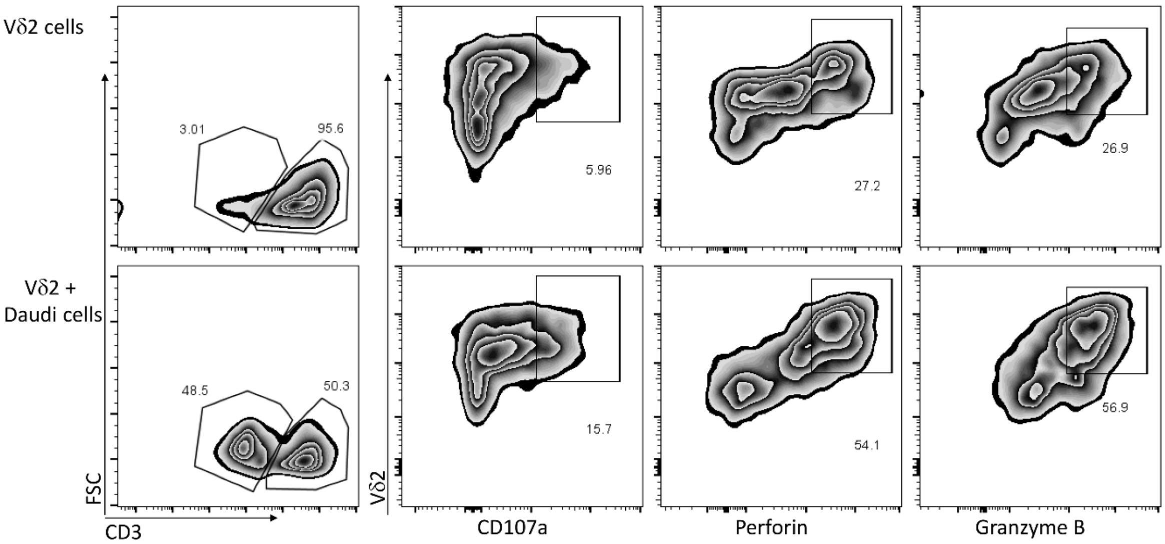

Figure S3. Representative zebra plots of degranulation (CD107a) and cytotoxicity markers perforin and Granzyme B in cultures of expanded isolated Vδ2 cells (top plots) and cocultures of Daudi cells and Vδ2 cells (1:1 ratio). Plots are derived from FSC/SSC and singlets gating.

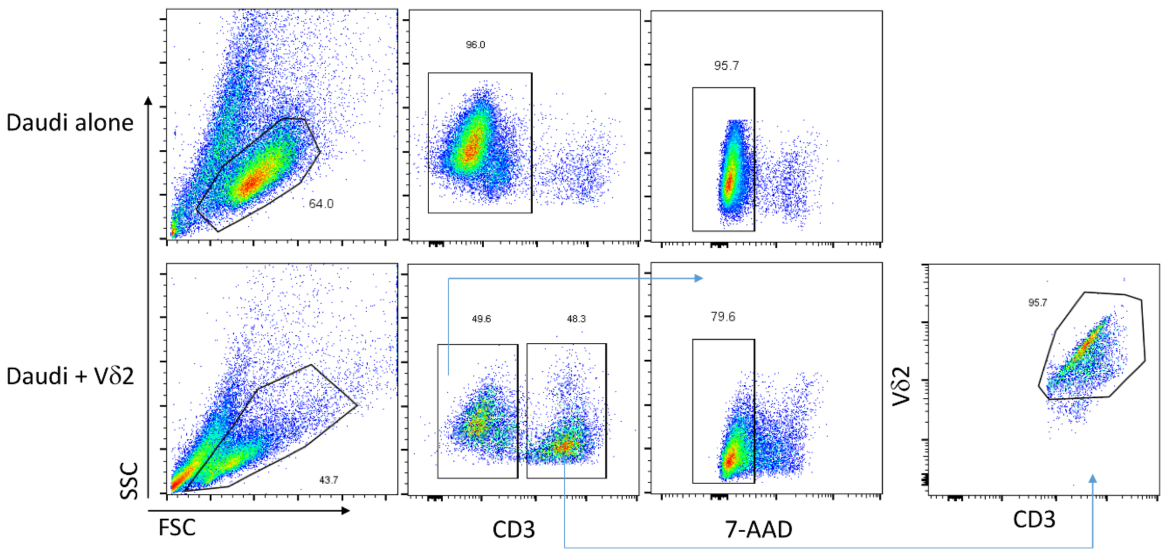

Figure S4. Representative pseudocolor plots showing killing of Daudi cells by expanded isolated Vδ2 cells. Frequency of Daudi cell death (CD3neg 7-AADneg cells) cultured alone (top) or in the presence of expanded, isolated Vδ2 cell (bottom) for 4 h. A 1:1 ra.
